# European Code Against Cancer, 5th edition – tobacco and nicotine containing products, second‐hand smoke, alcohol and cancer

**DOI:** 10.1002/1878-0261.70177

**Published:** 2026-01-16

**Authors:** Ariadna Feliu, Annie S. Anderson, Linda Bauld, Esteve Fernández, Michael Leitzmann, Sherry Morris, Bernard Srour, Constantine Vardavas, Ioana Vlad, Sabine Vuik, Matty Weijenberg, Rosa Alvarado‐Villacorta, Carlos Canelo‐Aybar, Hajo Zeeb, Joachim Schüz, Erica D'Souza, David Ritchie, Carolina Espina, Ioanna Bakogianni, Elio Riboli

**Affiliations:** ^1^ Environmental and Lifestyle Epidemiology Branch International Agency for Research on Cancer Lyon France; ^2^ Department of Primary Care and Public Health, School of Public Health Imperial College London London UK; ^3^ Division of Population Health & Genomics, Ninewells Medical School University of Dundee Dundee UK; ^4^ Usher Institute and Behavioural Research UK University of Edinburgh Edinburgh UK; ^5^ Tobacco Control Unit, WHO Collaborating Centre for Tobacco Control Catalan Institute of Oncology (ICO) L'Hospitalet de Llobregat Spain; ^6^ Tobacco Control Research Group Institut d'Investigació Mèdica de Bellvitge – IDIBELL L'Hospitalet de Llobregat Spain; ^7^ School of Medicine and Health Sciences, Campus de Bellvitge University of Barcelona L'Hospitalet del Llobregat Spain; ^8^ Department of Health Generalitat de Catalunya Barcelona Spain; ^9^ Department of Epidemiology and Preventive Medicine University of Regensburg Regensburg Germany; ^10^ Imperial College London London United Kingdom; ^11^ Université Sorbonne Paris Nord and Université Paris Cité INSERM, INRAE, CNAM, Center of Research in Epidemiology and StatisticS (CRESS), Nutritional Epidemiology Research Team (EREN) Bobigny France; ^12^ Nutrition and Cancer Research Network (NACRe Network) Jouy‐en‐Josas France; ^13^ Department of Hygiene, Epidemiology and Medical Statistics, School of Medicine National and Kapodistrian University of Athens Athens Greece; ^14^ Department of Oral Health Policy and Epidemiology Harvard School of Dental Medicine Boston MA USA; ^15^ Department of Policy and Public Affairs World Cancer Research Fund International London UK; ^16^ Organisation for Economic Co‐operation and Development Paris France; ^17^ Department of Epidemiology, GROW Research Institute for Oncology and Reproduction Maastricht University Maastricht The Netherlands; ^18^ Department of Clinical Epidemiology and Public Health Iberoamerican Cochrane Centre, Biomedical Research Institute Sant Pau Barcelona Spain; ^19^ Leibniz‐Institute for Prevention Research and Epidemiology – BIPS Bremen Germany; ^20^ Joint Research Centre – European Commission Ispra Italy; ^21^ Cancer Epidemiology and Prevention Research Unit Imperial College London London UK

**Keywords:** tobacco use, second‐hand smoke, alcohol consumption, cancer prevention, evidence‐based recommendations, European Code Against Cancer

## Abstract

Tobacco use, second‐hand tobacco smoke (SHS) exposure and alcohol consumption are well‐established carcinogens and major public health concerns. In the European Union (EU), tobacco and alcohol use are the leading preventable causes of cancer and four other major noncommunicable diseases (NCDs), significantly contributing to NCD‐related morbidity and mortality. Despite declining prevalence, consumption of these substances is still high in the region, especially among the most deprived. There is strong evidence that quitting smoking, minimising exposure to SHS and eliminating or reducing alcohol intake substantially lowers the risk of cancer. Comprehensive public health strategies at both the individual and population level are crucial to prevent cancer and other NCDs. Scientific evidence leads to two recommendations for individual action on tobacco in the European Code Against Cancer, 5th edition: (1) ‘Do not smoke. Do not use any form of tobacco, or vaping products. If you smoke, you should quit’; and (2) ‘Keep your home and car free of tobacco smoke’; and one on alcohol: (3) ‘Avoid alcoholic drinks’.

## Graphical abstract

The 5th edition of the European Code Against Cancer (ECAC5) contains 14 recommendations on cancer prevention. Here, we update the cancer prevention recommendations on tobacco, second‐hand smoke and alcohol listed as recommendations number 1, 2 and 6. Individuals are advised not to smoke or vape, to quit if they do, keep their home and car smoke‐free, and to avoid alcoholic drinks. Policymakers should strengthen control measures, including raising taxes, restricting sales and advertising, promoting public health campaigns and ensuring smoke‐free environments to reduce substance use and cancer risk.

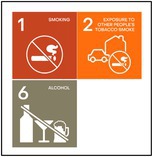

**Abbreviations**
€: Euro
CI: Confidence Intervals
COPD: Chronic Obstructive Pulmonary Disease
CVD: Cardiovascular disease
DNA: Deoxyribonucleic acid
EBCP: Europe's Beating Cancer Plan
EC: European Commission
ECAC: European Code Against Cancer
ECAC4: European Code Against Cancer, 4th edition
ECAC5: European Code Against Cancer, 5th edition
EU: European Union
FCTC: Framework Convention on Tobacco Control
HTPs: Heated Tobacco Products
IARC: International Agency for Research on Cancer
MS: member states
NCDs: Non‐communicable diseases
NRT: Nicotine replacement therapy
OR: Odds ratio
PAF: Population‐attributable fraction
SES: Socioeconomic status
SHA: Second‐hand aerosols
SHS: Second‐hand tobacco smoke
SIDS: Sudden infant death syndrome
TAPS: Tobacco advertising, promotion and sponsorship
WG: Working group
WHO: World Health Organization

## 1 Introduction

**Fig. 1:**
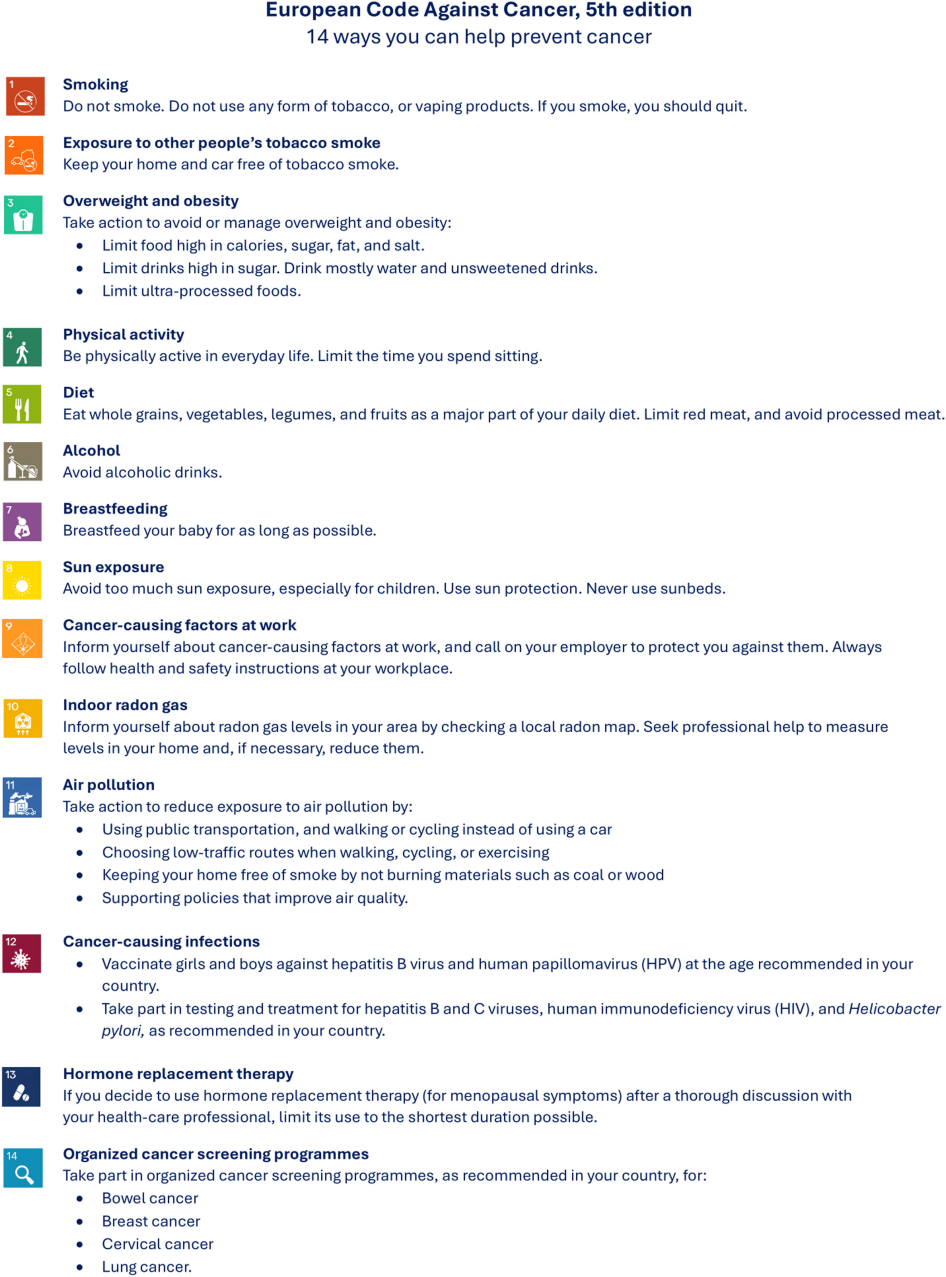
European Code Against Cancer, 5th edition: recommendations for individuals. The 14 recommendations of the European Code Against Cancer, 5th edition (ECAC5) adopted by the Scientific Committee of the ECAC5 project. © 2026 International Agency for Research on Cancer / WHO. Used with permission.

The European Code Against Cancer (ECAC) is a long‐standing set of evidence‐based recommendations developed by the European Union (EU) to help people reduce their risk of developing or dying from cancer. Its fifth edition (ECAC5) [1] (Fig. 1) builds on its predecessor (ECAC4) [2] with both coordinated by the International Agency for Research on Cancer (IARC/WHO) within the broader framework of the World Code Against Cancer Framework, launched by IARC in 2022 [3]. ECAC5 expands its target audience beyond individuals to also include recommendations for policymakers (Annex S1). This manuscript reviews and synthesises the latest scientific evidence on tobacco and nicotine‐containing products, second‐hand tobacco smoke (SHS) and alcohol in relation to cancer, using the IARC methodology described elsewhere [4]. In brief, a step‐by‐step decision‐making algorithm was conducted by a group of international experts, which includes systematic reviews and synthesis of evidence from epidemiological studies, intervention trials and other relevant research. The process also involved identifying public health priorities, evaluating the impact of interventions, including effects on equity and the feasibility, accessibility, and affordability for individuals, and engaging communities and stakeholders throughout. ECAC5 presents updated recommendations for the public, along with newly introduced population‐level policy recommendations designed to complement and reinforce individual‐level recommendations, accompanied by a summary of the supporting evidence.

Tobacco use, including exposure to SHS, and alcohol consumption are major public health concerns in the EU and well‐established risk factors for cancer. Tobacco and alcohol use are significant contributors to premature mortality and substantially increase the burden of disease in the region. They are key drivers of socioeconomic health inequities [5, 6], with mortality risks increasing as socioeconomic status (SES) declines. Given the strong evidence linking these risk factors to cancer [7], preventive public health interventions at both individual and population levels are critical to reduce the cancer burden in the EU attributable to them.

### 1.1 Prevalence of use of tobacco and nicotine‐containing products, exposure to second‐hand tobacco smoke and alcohol consumption within the EU

#### 1.1.1 Tobacco and nicotine‐containing products use

One‐fourth of people in the EU in 2023 were current smokers (men: 28% and women: 21%) and 20% were former smokers (men: 23% and women: 17%). Smoking prevalence in the EU varies widely across Member States (MS), ranging from 37% (Bulgaria) to 8% (Sweden). Significant differences also exist in the patterns of tobacco use across different social groups, being most prevalent among men and low‐SES groups [8]. On average, the prevalence of tobacco use is 5.4 percentage points higher among the most deprived compared to the least deprived segments of the population [9].

Although traditional manufactured cigarettes remain the most popular choice, other products are also widely used by people who smoke, such as roll‐your‐own tobacco, or, in certain countries, smokeless tobacco. Smokeless tobacco is highly prevalent in Sweden, where 46% of the people had used or tried this product in 2020 [10]. Over the past decades, novel tobacco and nicotine‐containing products (see Table 1, for types and definitions) have gained popularity, especially among youth [11]. Although current use of e‐cigarettes (also known as ‘vaping’, 3%) and heated tobacco products (HTPs) (2%) remains relatively low in the EU [8], many have already experimented with these products [12, 13]. Experimentation levels are significantly higher among the youth [13, 14] with 17% of students having tried e‐cigarettes [8]. Nicotine pouches are a relatively new product, similar to snus, that have only recently entered the market; consequently, regular use remains low across the EU (1%). However, 4% of EU residents report having used them and 2% having tried them. This product is the most popular among Scandinavian youth, especially in Sweden, where one‐fourth of people aged 15–24 are current users of nicotine pouches [8].

**Table 1:** Definition of novel tobacco and nicotine‐containing products.

Product	Definition
**Electronic cigarettes (e‐cigarettes)** (or Electronic Nicotine on Non‐Nicotine Delivery Systems [ENDS/ENNDS])	E‐cigarettes consist of a battery, an electrical heater and a liquid, that usually contains nicotine derived from tobacco, as well as flavourings, propylene glycol, vegetable glycerine, and other ingredients. This liquid is heated and aerosolized for user's inhalation [15, 16]
**Heated Tobacco Products (HTPs)** (or Heat‐not‐Burn)^a^	HTPs are nicotine delivery systems that consist of a small tobacco stick that is heated electronically, rather than burned, that generate an inhalable aerosol containing nicotine (and other toxicants) [17]
**Nicotine pouches**	These products provide nicotine in a substrate of white inert cellulose powder, flavourings, humectants, acidity regulators, and stabilisers. These pouches are placed between gum and lip where nicotine is absorbed through the oral mucosa [18]

a ‘Heat‐not‐Burn’ is a term popularized by the tobacco industry to market these products as safer alternatives to traditional cigarettes. Using ‘HTPs’ helps maintain objectivity and avoids endorsing industry‐driven narratives that may downplay potential health risks. The World Health Organization (WHO) and other health organizations use ‘Heated Tobacco Products’ to ensure clarity and consistency in public health communications and regulations.

#### 1.1.2 Exposure to second‐hand tobacco smoke and aerosols

Exposure to SHS, alternatively referred to as passive or involuntary smoking, has declined in the EU over the past decade [15], especially in the workplace, where exposure halved between 2005 and 2015 [16]. However, despite the early implementation of smoke‐free laws in most EU MS in public settings indoors [17], exposure remains high (23%). Prevalence of exposure is much higher outdoors, where smoke‐free policies are still lacking. Exposure to SHS outdoors was the highest in public spaces, such as parks or entrances to public buildings (78%), followed by terraces of hospitality venues (74%), open‐air public transportation stations (72%) and outdoor events (71%). Exposure in spaces dedicated to children and adolescents was lower (42%), but still concerningly high. SHS exposure varies between and within EU MS [8]. Men, youth and those from lower SES groups are more likely to be exposed to SHS [8, 18].

Exposure to SHS in private settings such as cars and homes is also concerning, especially among the most vulnerable populations: children and women [19]. Exposure to SHS in cars among youth varies widely across EU MS: 6% in Finland, 12% in Ireland, 15% in the Netherlands, 19% in Germany, 23% in Portugal, 36% in Belgium and 43% in Italy [20]. Regarding exposure to SHS at home, the prevalence of smoke‐free homes ranged from 44.4% in Greece to 79.4% in Ireland [21]. Similar to exposure levels in public settings, there is also a social gradient in exposure in private settings, as in all EU MS, exposure is higher among those from disadvantaged backgrounds [21].

E‐cigarettes also emit potentially hazardous compounds that reduce indoor air quality as they expose bystanders to quantifiable levels of particulate matter, key toxicants and contaminants [22], some of which are carcinogenic substances also found in other tobacco products [23]. In the EU, 16% of bystanders reported exposure to second‐hand aerosols (SHA) exhaled by e‐cigarette users in indoor settings, ranging from 4.3% (Spain) to 29.6% (England). Reported exposure was 6.4% in workplaces, 5.8% at home, 3.5% in public transport, 2.7% in cars and 8.3% in other indoor places (e.g. hospitality venues and leisure facilities) [24]. HTPs also emit SHA; however, evidence on exposure prevalence and aerosol composition remains scarce. Caution is therefore advised, as the full health effects of SHA exposure are still unclear.

#### 1.1.3 Alcohol consumption

Europe displays distinct drinking patterns influenced by geography and cultural traditions. While Southern European populations consume the most wine and have the lowest overall alcohol intake, Central European populations favour beer with minimal spirits consumption, and Eastern Europe exhibits the highest consumption of spirits, beer and total alcohol intake [25].

Despite decreasing alcohol consumption trends in the EU since the 1980s [26], 28.8% of adults consumed alcohol weekly and 22.8% monthly in 2019. In all EU MS, consumption was more frequent in men. Differences also exist by SES: daily use was the highest among the least educated (10.8%), while weekly use was higher among those with high education (38.3%) [27]. Moreover, 14.8% of the population engaged in heavy episodic drinking every month, with the highest prevalence (21.8%) observed in the 15–19 age group [28].

### 1.2 Cancer burden in the EU attributable to tobacco, second‐hand tobacco smoke and alcohol

There were 2.9 million new cancer cases and 1.3 million deaths in the EU in 2022 [29]. The cancer burden is estimated to increase by 25.3%, reaching 3.8 million new cases annually by 2050 [30]. Cancer is currently the second‐leading cause of death in the EU, with smoking and alcohol use being the top two risk factors [31].

#### 1.2.1 Tobacco products

Tobacco use is the leading preventable cause of cancer worldwide. Estimates suggest that smoking is responsible for approximately one‐third of all cancers [32]. In 2018, in Europe, 572 000 cancer cases in men and 186 000 in women were attributed to smoking, accounting for 28% and 10% of all cases, respectively. Lung cancer accounted for more than half of the total cancer burden attributable to smoking (382 000). Other major contributors to the total population‐attributable fraction (PAF) were lip, oral cavity and pharynx, bladder and laryngeal cancers in men (27% out of total PAF) and colorectal, pancreatic, oral cavity and pharyngeal cancers (21%) in women [33]. This burden is projected to significantly increase among vulnerable groups in the coming years, especially among disadvantaged women [34].

Lung cancer alone accounts for one‐fifth of global cancer‐related mortality, causing 1.8 million estimated deaths in 2022. Tobacco is the main risk factor [35], contributing to over 67% of lung‐cancer deaths globally [36]. Deaths from cancers caused by tobacco use globally increased from 1.5 million in 1990 to 2.5 million in 2019 for both sexes combined [37]. In the EU, smoking is responsible for approximately 700 000 deaths each year, of which over 256 468 are caused by cancer. Cancer‐related deaths were higher in men (194 613) than in women (61 855). While smoking‐attributable cancer deaths in men have decreased over the past decade, they have increased in women [38]. This difference may be attributed to the fact that smoking prevalence among women typically trails that of men by one to two decades. As a result, cancer deaths in women will continue to rise before eventually beginning to decline [39].

#### 1.2.2 Second‐hand tobacco smoke

SHS exposure poses a serious public health challenge due to its harmful effects on nonsmokers. Tobacco smoke contains thousands of chemicals and compounds, including many carcinogens, which, when inhaled, damage the human body and can lead to disease and death [40]. Many of the compounds in tobacco smoke are toxic and at least 69 of the 7000, including benzo[a]pyrene and N‐nitro dimethyl alanine, can cause cancer [7, 41].

SHS is a significant risk factor for lung cancer in never‐smokers, more frequently observed in women [42]. In the EU, between 16% and 24% of lung cancer cases in never‐smokers and long‐term former smokers are attributable to SHS exposure, mainly due to the contribution of work‐related exposure. Exposure to SHS during childhood is also associated with a relatively high proportion of lung cancers in adulthood [43].

SHS exposure was responsible for approximately 8850 cancer deaths in 2021 in Europe. Cancer‐related deaths due to SHS were nearly twice as high in men as in women. Similar to the trend observed in smoking‐related cancer deaths, SHS‐attributable deaths have decreased by 13.5% over the past decade, with steeper declines among men compared to women (16.2% vs. 7.5%) [38].

#### 1.2.3 Alcohol consumption

Alcohol consumption is one of the main known risk factors for cancer in the EU [44], only tobacco use causes more cancer cases. Europe contributes the highest global share of alcohol‐attributable cancer cases [45]. Of all new cancer cases in Europe in 2020, 4.5% were estimated to be attributable to alcohol drinking [46]. In the EU, alcohol consumption was estimated to cause 111 300 new cases of cancer (4.1% of all new cases) in 2020. Almost 70% of these cases were in men, partly because they drink about three times as much alcohol as women [47].

There is no safe level of alcohol consumption, as even low intake levels are associated with an increased risk of cancer [7]. In 2017, light‐to‐moderate alcohol consumption contributed to approximately 23 000 new cancer cases in the EU, accounting for 13.3% of all alcohol‐attributable cancers [48]. Nearly half of these cases were female breast cancers. Over one‐third of alcohol‐related cancers at these consumption levels were associated with drinking less than one standard drink per day (total: 37%; women: 40%; men: 32%) [48]. Yet, population risk perception remains low; for example, 23.5% of the French population believes that drinking a little wine generally lowers cancer risk compared with drinking none [49].

## 2 Recommendations for individuals

The ECAC5 Lifestyle Determinants Working Group (WG) reviewed the most recent evidence on tobacco, SHS and alcohol, and their associations with cancer. Using IARC methodology [4], the WG updated the ECAC4 recommendations, which required at least *sufficient evidence* to demonstrate a reduction in cancer risk. In addition to the strength of the evidence, the WG evaluated each recommendation for equity, suitability, actionability and acceptability within the EU context.

### 2.1 Scientific justification for updating the recommendations in ECAC5

#### 2.1.1 Tobacco‐ and nicotine‐containing products

##### 2.1.1.1 Evidence on the association between tobacco use and cancer

Tobacco is an established Group 1 carcinogen as classified by IARC. Tobacco use is associated with 16 different types of cancers [7]. Other forms of tobacco, such as smokeless tobacco, also have been proven to cause cancers of the oral cavity, oesophagus and pancreas [7], as reported by Leon *et al*. [50] in ECAC4.

HTPs, although noncombustible, contain tobacco and are therefore classified by both the EU [51] and the WHO [52] as tobacco products, or ‘other forms of tobacco’ in line with ECAC terminology, which also includes smokeless tobacco. HTPs incorporate novel features and designs intended to replicate the experience of smoking conventional cigarettes and to appeal to new users [53]. There is currently no conclusive evidence that HTPs are less harmful than conventional cigarettes. On the contrary, certain harmful chemicals have been detected in higher concentrations in HTP emissions compared to conventional cigarette smoke. Moreover, HTPs produce novel substances, including metals and volatile organic compounds, not typically generated by conventional cigarettes, whose health impacts remain unassessed and may pose additional toxicological risks [14, 54]. Although the long‐term health effects of HTPs use are still unknown due to the lack of epidemiological data to evaluate the chronic disease risks associated with these products, there is growing evidence of adverse health effects on the cardiovascular and respiratory systems [55].

Nicotine‐containing products, such as e‐cigarettes and nicotine pouches, are often marketed as ‘safer’ alternatives to conventional combustible cigarettes, despite inconclusive evidence on their long‐term health risks. These products generally produce lower levels of certain carcinogens and toxic chemicals than traditional tobacco products (e.g. manufactured cigarettes). Nevertheless, they are not without risk [14]. E‐cigarettes use (or vaping) has been linked in some studies to an increased risk of myocardial infarction and stroke among daily users, as well as asthma exacerbation and chronic bronchitis [56]. Long‐term effects of e‐cigarette use, however, are still uncertain, including cancer data. A recent evidence review concluded that there is some but insufficient evidence that vaping alters gene expression and DNA methylation [57].

Despite the lack of evidence linking e‐cigarette use directly to cancer, studies suggest that vaping may be associated with smoking initiation among nonsmokers. Following the IARC methodology for modifying existing ECAC4 recommendations or introducing new ones [4], the ECAC5 WG on Lifestyle Determinants revised a recent systematic review conducted as part of a previous project within the World Code Against Cancer Framework [58]. The aim was to evaluate whether e‐cigarettes may serve as a gateway to the use of combustible cigarettes or other tobacco products among young people. To update this evidence‐base for ECAC5, the WG commissioned a new systematic review to address the research question ‘*Are electronic cigarettes a gateway to the use of combustible cigarettes or other tobacco products among young people*?’ [59]. The review included studies assessing the association between e‐cigarettes use, with and without nicotine, and smoking initiation in youth (see Table 2, for PECO question).

**Table 2:** Structured clinical question: Population, Exposure, Control and Outcome (PECO). HTPs, Heated Tobacco Products

Population	Exposure	Control	Outcomes
People aged between 10 and 29 years who have never consumed tobacco products	E‐cigarettes with and without nicotine (including menthol and flavoured cigarettes); considering different levels of exposure: Experimentation (ever) and regular use	No exposure	Smoking initiation with: Combustible cigarettes and HTPs Combustible cigarettes and e‐cigarettes Combustible cigarettes (exclusive) HTPs (exclusive)

**Fig. 2:**
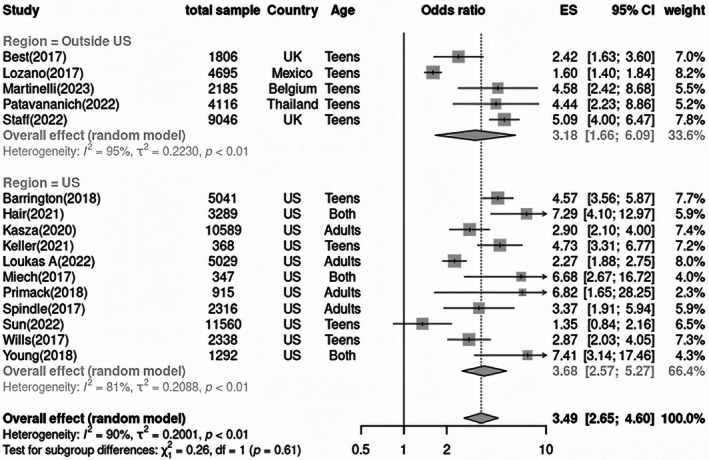
Effect of ever compared to never use of e‐cigarettes in reporting initiation of traditional tobacco use. The effect size (ES) corresponds to the Odds Ratio (OR) for each study. Each square represents the point estimate (ES/OR) for each individual study, and the error bars through it represent the 95% Confidence Intervals (CI). The diamond at the bottom represents the pooled effect size (summary OR) from the meta‐analysis under a random‐effects model. UK, United Kingdom; US, United States.

Findings suggest that both ever and regular use of e‐cigarettes among young people are associated with over threefold higher odds of initiating smoking combustible cigarettes (adjusted odds ratio (aOR) = 3.49, 95% Confidence Intervals (CI): 2.65–4.60) (Fig. 2), thereby increasing their probability of becoming smokers and suffering from tobacco‐related harms in the medium and long run [54]. A sensitivity analysis of the effect of ever compared to never use of e‐cigarettes adjusted for SES confirmed the results (aOR = 3.47, 95% CI: 2.40–5.02). Despite moderate certainty due to potential biases in the included studies, these findings indicate a significant public health concern regarding the role of e‐cigarettes as a gateway to traditional smoking among never smokers.

##### 2.1.1.2 Benefits for cancer prevention of following the tobacco recommendation

Smoking initiation and progression occur predominantly during adolescence and young adulthood [60]. Individuals who remain smoke‐free through their mid‐20s are unlikely to uptake smoking later in life. Hence, preventing tobacco use among youth remains a key strategy to end the tobacco epidemic [36]. Evidence from behavioural and biological studies suggests that adolescents and young adults are particularly vulnerable to nicotine addiction [61]. Moreover, the majority of adults who smoke report regretting having started smoking [62], which highlights the importance of early prevention in reducing long‐term consequences of tobacco use.

Quitting smoking at any age has been proven to yield health benefits, including substantial improvements in cardiovascular health and lung function [63]. Smoking cessation is proven to reduce the risk for many adverse health effects, including cancer [64]. Adults over 35 years continuing to smoke face a substantial reduction in life expectancy. Quitting smoking as early as possible is important as it leads to gains in life expectancy as compared to continuing to smoke [65]. Cessation pre‐operatively [66] or before treatment after cancer diagnosis [67] improves recovery and may improve survival. However, almost half of the people who smoke in the EU have never tried to quit and most of them do not intend to do so in the next 6 months [68].

Despite most people who smoke quit without professional help [69, 70], evidence suggests that chances of long‐term smoking cessation increase when using evidence‐based smoking cessation interventions [71]. Behavioural support and pharmacotherapy, such as bupropion, varenicline, cytisine or nicotine replacement therapy (NRT), are safe and effective interventions for smoking cessation. These drugs are effective when used alone but combining them yields the highest success in smoking cessation [72, 73]. Nicotine‐containing products, particularly e‐cigarettes, have also been proposed as a smoking cessation strategy. Evidence of their effectiveness in supporting quitting is growing. A recent Cochrane review [74] reported that e‐cigarettes may help people stop smoking for at least 6 months; however, the authors concluded that more evidence is needed, as results were based on a limited number of studies for most outcomes.

##### 2.1.1.3 Equity, suitability, actionability and acceptability

Tobacco use significantly contributes to health and mortality inequities [5, 6]. Smoking is the leading preventable cause of health inequities in the EU [75], as people from more deprived groups tend to smoke more frequently and have higher addiction levels [76]. Socio‐economic disadvantage also plays an important role in smoking cessation outcomes. People who smoke from low SES often report lower self‐efficacy to quit, intention to quit, quit attempts and quit success [77, 78, 79]. Health inequities also exist in access to smoking cessation services, often due to lack of awareness [80].

Although the availability of tobacco cessation services varies across EU MS, most already provide some level of support [69], including but not limited to operating a national quitline and providing cost coverage of NRT and other cessation services [41]. Offering cessation services requires an initial investment from MS; however, these interventions are highly cost‐effective and feasible [81]. For example, in Germany, while smoking cessation medications and behavioural support were estimated to cost €220 and €9 per recipient, respectively, the estimated annual cost of lung cancer treatment per case was €52 106 [82].

Public acceptability of smoking restrictions is high and has increased over time [83], including among people who smoke and those from more deprived backgrounds, although support in these groups tends to be lower [84]. Social norms are associated with increased quitting‐related cognitions and behaviours. People who smoke who endorse stronger nonsmoking norms are more motivated to stop smoking and more likely to make a quit attempt, with no differences across social groups [85]. Stronger educational efforts in communicating health risks of smoking and promoting quit attempts are needed, as significant gaps, especially among low‐SES groups, still exist in understanding of these risks among people who smoke [86].

##### 2.1.1.4 Presentation of the recommendation

In view of the above, the ECAC4 recommendation, ‘*Do not smoke. Do not use any form of tobacco’* was updated to:

Do not smoke. Do not use any form of tobacco, or vaping products. If you smoke, you should quit. (Fig. 1).

The revised recommendation, aligned with the Latin America and the Caribbean Code Against Cancer, 1st edition [58], includes e‐cigarettes, as these novel nicotine‐containing products have become increasingly popular among youth. Recent evidence suggests that vaping among nonsmokers may act as a gateway to tobacco smoking. Additionally, the updated recommendation explicitly encourages individuals who already smoke to quit, as quitting at any age significantly reduces cancer risk and improves health outcomes. The WG decided to keep the final statement on cessation sufficiently broad to ensure that people who smoke were not discouraged from using e‐cigarettes as a cessation aid.

#### 2.1.2 Exposure to second‐hand tobacco smoke

##### 2.1.2.1 Evidence on the association between SHS and cancer

Exposure to SHS, which is the combination of tobacco smoke exhaled by others (i.e. mainstream smoke) and that coming from the burning end of combustible tobacco products (i.e. side stream smoke), is a well‐established carcinogen that increases the risk of overall cancer for never smokers [87]. IARC has classified SHS as a cause of lung cancer (Group 1 carcinogen) and a possible cause of cancers of the larynx and pharynx [7]. Moreover, SHS also increases the risk of breast cancer in never‐smoking women, particularly among premenopausal women [88, 89], and there is sufficient evidence that parental smoking causes hepatoblastoma in children, according to the latest IARC Monograph [7].

There is a 24% overall excess risk of lung cancer among never‐smokers who were exposed to SHS compared with those who were not exposed [42]. There is also a dose–response effect as the risk of lung cancer increases with the intensity, duration and pack‐years of SHS exposure. There is no safe level of exposure, as even brief exposure has been proven to be harmful for health [7].

##### 2.1.2.2 Benefits for cancer prevention of following the SHS recommendation

Children suffer disproportionately from SHS exposure as, unlike adults, they often have little to no control over their exposure to tobacco smoke. Parental smoking at home and in cars is a major source of exposure, as children generally spend a significant amount of time at home [20, 90]. Over 4 500 000 disability‐adjusted life‐years are estimated to be attributed to SHS among children younger than 14 years [91].

Exposure to SHS also increases the difficulty of maintaining abstinence, hindering smoking cessation success since smoking environments may trigger smoking‐related cues and social pressures [92]. Exposure to SHS at home is also a significant barrier to quitting smoking, even among patients with cancers not traditionally linked to tobacco [93]. This evidence highlights the importance of promoting smoke‐free environments, especially in private settings, to reduce smoking initiation, especially among youth, and to support cessation and long‐term abstinence [94, 95].

##### 2.1.2.3 Equity, suitability, actionability and acceptability

Support for smoke‐free measures is strong in many countries [96], including public support for novel smoke‐free policies in outdoor and private areas, particularly in places frequented by children [97]. A previous study in six EU MS reported that one in four (27%) of people who smoked had a total smoking ban in their home and 61% in cars. Although prevalence was lower among the low‐educated group, many had already implemented total or partial smoking bans [98].

Smoke‐free environments have been proven to be effective in reducing exposure to SHS and yield significant public health benefits [99]. Although most smoke‐free laws in the EU do not cover cars and homes, their implementation is associated with an increase in the percentage of smoke‐free homes [100, 101], likely reflecting a reduction in the social acceptability of tobacco use. While maintaining a smoke‐free environment is not always fully within the control of those exposed, raising awareness about the risks of SHS may encourage broader adoption of comprehensive smoke‐free rules [102].

##### 2.1.2.4 Presentation of the recommendation

Considering the above, the ECAC4 recommendation *‘Make your home smoke free. Support smoke‐free policies in your workplace’*, was updated in ECAC5 to:

Keep your home and car free of tobacco smoke. (Fig. 1).

The main change is a shift in focus to exposure in private settings (i.e. homes and cars). The updated recommendation adds cars as a key source of SHS exposure, particularly for children whose parents smoke, highlighting the importance of smoke‐free environments in settings where most EU MS still lack regulation and where protection relies on individual action. The recommendation for the individual no longer includes supporting workplace smoke‐free policies, as ECAC5 includes recommendations directly targeted to policymakers [8].

#### 2.1.3 Alcohol consumption

##### 2.1.3.1 Evidence on the association between alcohol and cancer

Alcohol consumption is also a well‐established Group 1 carcinogen [7] as there is sufficient evidence that it causes cancers of the oral cavity, pharynx, larynx, oesophagus, gastric, colorectum, liver (hepatocellular carcinoma) and female breast [103]. All types of alcoholic drinks, including beer, wine and spirits, can cause cancer, with the risk beginning at low levels and increasing significantly as both intake and ethanol content rise [7, 104]. In fact, there is no safe level of alcohol consumption since there is no threshold below which there is no risk increase for at least some cancers [103]. Strong evidence also exists of the multiplicative increase in the risk of cancer of the upper aero‐digestive tract (mouth, tongue, pharynx, larynx and oesophagus—squamous cell) and liver for those people who smoke and drink [105].

Since the publication of the last edition of the ECAC [106], new research has emerged regarding the potential effect of light to moderate alcohol consumption on the risk of cardiovascular disease (CVD). Recent findings from well‐designed prospective cohort studies suggest that the cardiovascular benefits of alcohol have been overestimated in the past [107]. One recent cohort study found that alcohol consumption, regardless of the amount, is associated with an increased cardiovascular risk, with a more pronounced risk observed at higher consumption levels [108]. There is also evidence from Mendelian Randomisation studies of a causal relationship between higher alcohol consumption and increased risk of stroke and peripheral artery disease [109]. In the light of this evidence, not drinking alcohol is the healthiest choice, as no safe amount of alcohol consumption can be established [110].

##### 2.1.3.2 Benefits for cancer prevention of following the alcohol recommendation

There is strong evidence that reducing alcohol consumption or stopping drinking completely decreases the risk of oral cancer and oesophageal cancer [111]. A recent cohort study found that individuals who reduced their consumption levels from heavy to moderate or light levels and those who maintained long‐term abstinence had a decreased risk of alcohol‐related and all cancers [112].

##### 2.1.3.3 Equity, suitability, actionability and acceptability

Alcohol use is a major driver of health and mortality disparities [5]. Although individuals from disadvantaged groups may consume the same amount or even less alcohol than those from higher SES, they experience disproportionately higher rates of alcohol‐related hospital admissions and deaths [113]. This reflects not only differential vulnerability but also inequities in access to health care, early intervention and support services.

Moreover, cultural perceptions of alcohol consumption may also affect the acceptability of the recommendation. While high levels of alcohol use are often viewed as unacceptable, moderate consumption frequently remains socially acceptable, or even encouraged, in many contexts [114]. Therefore, public policies at denormalising alcohol consumption are necessary to foster cultural acceptance of measures that promote reducing alcohol intake to zero.

##### 2.1.3.4 Presentation of the recommendation

Given the above, the 5th edition of the ECAC has updated the previous recommendation, ‘*If you drink alcohol of any type, limit your intake. Not drinking alcohol is better for cancer prevention.’* to:

Avoid alcoholic drinks (Fig. 1)

This aligns with the Latin America and the Caribbean Code Against Cancer, 1st edition [58], as the latest evidence indicates that, for cancer prevention, the best option is to avoid drinking alcohol completely, since no level of consumption is considered risk free.

### 2.2 Cobenefits for prevention of non‐communicable diseases other than cancer with similar risk factors and opportunities for health promotion

Noncommunicable diseases (NCDs) are responsible for about 60% of global deaths and are largely preventable through lifestyle changes [115]. The main risk factors for NCDs are well known. Tobacco use, including exposure to SHS, and alcohol consumption are common risk factors for four major NCDs, including cancer, causing one in six of all NCD‐related deaths [116].

Smoking is the leading cause of CVD, including heart disease, stroke and peripheral artery disease; and of chronic respiratory diseases, such as chronic obstructive pulmonary disease (COPD), emphysema and chronic bronchitis. It is also directly linked to the development and progression of many chronic health conditions [117]. Quitting smoking is the most efficient intervention to reduce the risk of NCDs and improve overall health [118].

Exposure to SHS can also cause CVD, including heart disease and stroke, and respiratory conditions, including COPD, in never‐smokers. Indeed, it has been estimated that SHS increases the risk of ischemic heart disease by at least 8%, stroke by 5% and type 2 diabetes by 1% [119]. Among children, maternal smoking and SHS exposure during pregnancy are detrimental to fetal growth and development, leading to adverse birth outcomes and perinatal and infant mortality [120]. Exposure to SHS can have immediate and long‐term effects on children's health, including increased risk of asthma, chronic bronchitis, coughing and wheezing, and sudden infant death syndrome (SIDS) [119]. Reducing or avoiding exposure to SHS, especially protecting children, will also contribute to reducing the risk of these diseases and protecting children's health and development.

Finally, alcohol consumption has also been causally associated with about 60 types of diseases, including heart disease, stroke and vascular diseases, liver cirrhosis, birth defects and intellectual impairments. Alcohol also contributes to death and disability through accidents and injuries, assault, violence, homicide and suicide [106]. Hence, avoiding drinking alcohol and reducing the amount consumed has numerous other benefits for overall health and well‐being, besides reducing the risk of cancer.

## 3 Recommendations for policymakers

Cancer is a leading cause of morbidity and mortality in the EU, with many of its risk factors preventable through effective policies. Public health action through effective evidence‐based strategies is key to reducing cancer incidence as well as preventing the negative health effects and social harm caused by tobacco and nicotine‐containing products and alcohol use.

The set of evidence‐based policy recommendations for policymakers on tobacco, nicotine‐containing products, SHS and alcohol consumption proposed by the WG on Lifestyle Determinants as part of the ECAC5 (Tables 3, 4, 5) are aimed at enabling environments in which individuals can adopt the recommendations in Fig. 1. These policy recommendations are based on existing evidence‐based international authoritative policies and were selected according to the IARC methodology and prioritised using the Nuffield Ladder of Interventions [4, 121], and are supported in part by Europe's Beating Cancer Plan (EBCP) [122] and the WHO NCD best buys [123].

**Table 3:** European Code against Cancer, 5th edition: recommendations for policymakers on tobacco‐ and nicotine‐containing products.

Tobacco‐ and nicotine‐containing products
**Adopt, implement, and enforce comprehensive tobacco control policies, as per the WHO Framework Convention on Tobacco Control, including**
○ Measures to raise tobacco taxes to at least 75% of tobacco's retail price and significantly increase tobacco taxes every year. All tobacco products should be taxed in a comparable way as appropriate, in particular where the risk of substitution exists.
○ Measures to restrict the availability and accessibility of tobacco products. This includes increasing the age of sale and allowing the sale of tobacco products only in licensed stores. ○ Measures to ban tobacco advertising, promotion, and sponsorship, including display bans at the point of sale.
○ Provision of smoking cessation services. Identify and allocate sustainable funding for tobacco cessation and tobacco dependence treatment programmes.
○ Large graphic health warnings, labelling, and plain, standardized packaging for tobacco products.
**Extend such regulations to apply to all tobacco products, electronic cigarettes, and all novel tobacco‐ and nicotine‐containing products.**
**Establish and work towards achieving a goal for a tobacco‐free generation in your country.**
**Complementing the above‐mentioned policy measures, implement regular public health campaigns to raise awareness of the damaging effects of tobacco and the benefits of smoking cessation.**

© 2026 International Agency for Research on Cancer / WHO. Used with permission. References: • World Health Organization Framework Convention on Tobacco Control. Geneva: WHO; 2003. Available from: https://apps.who.int/iris/rest/bitstreams/50793/retrieve [127]. .• Directive 2014/40/EU of 3 April 2014 on the approximation of the laws, regulations and administrative provisions of the Member States concerning the manufacture, presentation and sale of tobacco and related products and repealing Directive 2001/37/EC. *OJEU*. 2014;**L127**:1–38. Available from: https://eur‐lex.europa.eu/eli/dir/2014/40/oj [51]. .• Council Directive (EU) 2020/262 of 19 December 2019 laying down the general arrangements for excise duty (recast). *OJEU*. 2020;**L58**:4–21. Available from: https://eur‐lex.europa.eu/eli/dir/2020/262/oj [146]. .• Directive 2010/13/EU of 10 March 2010 on the coordination of certain provisions laid down by law, regulation or administrative action in Member States concerning the provision of audiovisual media services (Audiovisual Media Services Directive). *OJEU*. 2010;**L95**:1–24. Available from: https://eur‐lex.europa.eu/eli/dir/2010/13/oj [132].

### 3.1 Tobacco‐ and nicotine‐containing products and exposure to second‐hand tobacco smoke

Effective tobacco control policies have been successful in decreasing smoking prevalence worldwide [124] and in the EU [125], consequently reducing tobacco‐attributable morbidity and mortality [126]. Substantial efforts have been made to prevent and control the use of tobacco‐ and nicotine‐containing products globally. These efforts to address the tobacco epidemic culminated in the adoption in 2003 of the WHO Framework Convention for Tobacco Control (FCTC). The WHO FCTC is a legally binding treaty aimed at *‘[…] protecting present and future generations from the devastating health, social, environmental and economic consequences of tobacco consumption and exposure to tobacco smoke’* [127].

To assist Parties in effectively fulfilling their obligations under the WHO FCTC, the WHO introduced the MPOWER strategy, a package of six evidence‐based policy measures designed to reduce tobacco use [128]. These measures include monitoring tobacco use and prevention policies, protecting people from tobacco smoke, offering help to quit tobacco use, warning about the dangers of tobacco, enforcing bans on tobacco advertising, promotion and sponsorship (TAPS), and raising taxes on tobacco to make them less affordable (Table 6).

Although the EU and each of its MS have ratified the WHO FCTC, the level of tobacco control policy implementation and enforcement varies widely across countries [129]. Regional progress in tobacco control has also been driven by EU‐level actions, including several directives: tobacco products regulations [Tobacco Products Directive [51]] to govern the manufacture, presentation and sale of tobacco and related products; on excise duties applied to manufactured tobacco [Tobacco Taxation Directive [130]]; and on cross‐border tobacco advertising and sponsorship [Tobacco Advertising Directive [131]]. Additionally, the Audio‐visual Media Services Directive [132] bans TAPS in all audiovisual commercial communications forms, including product placement.

In line with the WHO FCTC and EU Directives, ECAC5 recommends raising excise taxes and prices annually to reach at least 75% of tobacco's retail price, making products progressively less affordable, as this is the most effective and cost‐effective measure for reducing tobacco use [133]. It also encourages MS to enforce TAPS bans to protect individuals, particularly youth, from marketing tactics; and to strengthen labelling policies, ensuring health pictorial warnings are more prominent (Table 3). ECAC5 advocates for plain packaging, which removes logos, colours, and promotional information, leaving only brand and product names in a standard font. This measure has been implemented in a number of European countries and has proven to be effective in reducing the appeal of smoking [134].

The EU has recently reviewed the Council Recommendation on smoke‐free environments to better protect people from the effects of SHS and SHA, particularly children and young people. This new initiative recommends that MS extend smoke‐free laws to key outdoor areas, and to include emerging products (i.e. e‐cigarettes) [135]. Evidence shows that smoke‐free laws in public settings can also lead to voluntary smoking bans in homes by shifting attitudes and social norms towards SHS exposure [101]. On this note, ECAC5 encourages MS to ensure that all indoor public settings are covered by smoke‐free laws without exemptions and that compliance is high. It also recommends extending this legislation to outdoor public settings and including all tobacco‐ and nicotine‐containing products, as recommended by the EU. Additionally, it advocates for national public health campaigns to raise awareness about the health risks of tobacco use, as well as exposure to SHS and SHA, and to promote smoke‐free cars and homes to protect bystanders, particularly children (Table 4).

**Table 4:** European Code Against Cancer, 5th edition: recommendations for policymakers on second‐hand tobacco smoke.

Second‐hand smoke	
•	Enforce legislation to eliminate exposure to second‐hand tobacco smoke in all indoor workplaces, public places, and transportation
•	Extend smoke‐free laws to outdoor public places, in particular health‐care centres and areas where children and adolescents could be exposed, such as educational settings and playgrounds
•	Extend smoke‐free legislation to include all novel tobacco and nicotine‐containing products
•	Complementing the above‐mentioned policy measures, implement regular smoke‐free environment campaigns for private settings, such as homes and vehicles, and regular public health campaigns to raise awareness of the effects of exposure to second‐hand smoke on health and the risk of cancer

© 2026 International Agency for Research on Cancer / WHO. Used with permission. References: • World Health Organization Framework Convention on Tobacco Control. Geneva: WHO; 2003. Available from: https://apps.who.int/iris/rest/bitstreams/50793/retrieve [127]. • Council Recommendation of 3 December 2024 on smoke‐ and aerosol‐free environments replacing Council Recommendation 2009/C 296/02. *OJEU*. 2024;**C7425**:1–5. Available from: https://eur‐lex.europa.eu/eli/C/2024/7425 [135].

[Correction added on 24 January 2026: The table body has been updated.]

Through the EBCP, the EU has committed to achieving a ‘Tobacco‐Free Generation’ by 2040. This strategy is part of the ‘endgame’ measures aimed at reducing smoking prevalence to minimal levels (< 5%). Other proposed policies to reach this goal include prohibiting tobacco sales for anyone born after a certain year [136] and raising the legal age of sale above 18. This measure has already been approved in Ireland (age of sale 21) and is under consideration in other MS, including Finland, the Netherlands and Slovenia [137]. In this regard, ECAC5 encourages MS to set their own targets for a tobacco‐free generation and to raise the legal minimum age of sale to prevent youth initiation by restricting the availability and accessibility of tobacco products.

Certain policy areas, such as smoking cessation treatment, remain under the exclusive competence of MS. Smoking cessation treatment should be delivered in both primary and specialty care across all out‐ and inpatient settings [68]. However, integrating these practices into routine clinical care and infrastructures presents challenges, including a lack of standardised protocols, limited training of clinical staff and financial constraints [138]. The availability of professional cessation support varies across MS. While most operate national toll‐free quitlines, only about one‐third provide subsidised access to NRT [69]. Therefore, ECAC5 calls on MS to strengthen national tobacco cessation services by integrating brief interventions into primary and specialised‐care settings, ensuring cost‐covered pharmacotherapy through the healthcare system, and offering national toll‐free quitlines to reach all populations, particularly the most deprived.

Regarding emerging products, such as e‐cigarettes, the WHO has urged countries to take action to prevent their uptake [139]. These measures include regulating e‐cigarettes to reduce their appeal and harm; protecting the public from misleading claims; prohibiting sales to children; applying WHO FCTC tobacco control measures to e‐cigarettes; and raising awareness about associated health risks. Following WHO recommendations, ECAC5 also calls for action to ensure that tobacco control policies are extended to all tobacco‐ and nicotine‐containing products.

Tobacco control policies are highly cost‐effective in all levels of implementation, as even in the most conservative scenarios with only a 1% relative smoking prevalence reduction, policies remain highly cost‐effective [140]. Therefore, a comprehensive approach incorporating all six tobacco control measures can generate significant public health benefits and economic savings, as healthcare cost savings outweigh the cost of interventions [141].

### 3.2 Alcohol consumption

Integrated policy approaches aimed at reducing alcohol consumption and denormalising its use include taxation, age restrictions, regulating marketing and advertising. Interventions in primary care and community settings have also proven to reduce alcohol‐related harm [142]. MS' governments and local administrations have implemented various measures to regulate the production, sale and consumption of alcoholic beverages, as well as to address alcohol‐related problems. However, the level of alcohol policy implementation varies widely across the EU [143].

For example, while most MS have licencing systems to control the production and/or sale of alcoholic beverages, only 16 out of the 27 restrict opening hours and days of sales. Similarly, differences exist in the minimum legal purchasing age: while all MS have established a legal minimum age, it ranges from 16 to 20 years, with variations depending on the type of product or the context of consumption (e.g. on‐premises vs. off‐premises; or whether the consumer is accompanied by an adult) [144].

Although EU MS retain primary responsibility for their national alcohol policies, the EU plays a regulatory role in specific areas. The EU sets harmonised minimum excise duty rates for alcohol and alcoholic beverages through Directive (EU) 2020/1151 [145] and Directive (EU) 2020/262 [146] and regulates food information requirements through Regulation (EU) 1169/2011 [147], including wine Regulation (EU) 2021/2117 [148] In particular, the EU prohibits health claims on alcoholic beverages exceeding 1.2% alcohol by volume to enhance consumer protection.

The European Commission (EC)'s EBCP [122] recognises alcohol‐related harm as a major public health concern and set a target of at least a 10% relative reduction in harmful alcohol use by 2025. Through the EBCP, the EC has committed to reviewing EU legislation on alcohol taxation and cross‐border alcohol purchases by private individuals; regulating alcohol promotion; introducing health warning labels; and supporting MS in implementing evidence‐based brief interventions on alcohol consumption.

At the global level, several organisations have issued policy recommendations to address alcohol‐related harm, including the *WHO Global Alcohol Action Plan 2022–2030* [149]. In collaboration with the United Nations, the WHO launched the *SAFER* initiative [150] to support countries in reducing harmful alcohol use by strengthening the implementation of the *Global Strategy to Reduce the Harmful Use of Alcohol* [151]. This initiative focusses on five evidence‐based cost‐effective alcohol policy interventions: (1) strengthening restrictions on alcohol availability; (2) advancing and enforcing drink‐driving countermeasures; (3) facilitating access to screening, brief interventions, and treatment; (4) enforcing bans or comprehensive restrictions on alcohol advertising, sponsorship, and promotion; and (5) increasing alcohol prices through excise taxes and other pricing policies.

In line with EU and WHO recommendations, ECAC5 calls on MS to increase domestic alcohol taxes, establish minimum pricing and restrict the availability and accessibility of alcoholic beverages through licencing systems and sales limitations. Additionally, it encourages raising the legal minimum age of sale to prevent sales to minors [152], banning or restricting alcohol advertising, introducing health warning labels on alcohol packaging, ensuring that alcohol is not available in public catering services, and promoting public health campaigns to raise awareness of alcohol's health risks, particularly its association with cancer. Further measures include facilitating access to screening, brief interventions and treatment, as well as increasing public health system capacity to support prevention and treatment efforts, particularly for vulnerable groups (Table 5).

**Table 5:** European Code Against Cancer, 5th edition: recommendations for policymakers on alcohol.

Alcohol	
•	Increase prices of alcohol through taxation to make alcohol less affordable
•	Establish a minimum price for all alcoholic beverages
•	Restrict the availability and accessibility of all alcoholic beverages
•	Ensure that no alcoholic beverages are offered in any public catering services
•	Increase minimum legal age limits to at least 19 years for selling and serving all alcoholic beverages
•	Ban or restrict advertising, promotion, and sponsorship of all alcoholic beverages in all media and for all purposes, especially those targeting minors
•	Facilitate access to screening, brief interventions, and treatment of alcohol use disorder in primary care and community settings
•	Introduce health warning labels related to alcohol consumption and nutrition labelling on all alcoholic beverages
•	Complementing the above‐mentioned policy measures, implement regular public health campaigns to raise awareness of the detrimental effects of alcohol intake on health and its association with cancer risk

© 2026 International Agency for Research on Cancer / WHO. Used with permission. References: • Council Directive (EU) 2020/262 of 19 December 2019 laying down the general arrangements for excise duty (recast). *OJEU*. 2020;**L58**:4–21. Available from: https://eur‐lex.europa.eu/eli/dir/2020/262/oj [146].

• Council Directive (EU) 2020/1151 of 29 July 2020 amending Directive 92/83/EEC on the harmonisation of the structures of excise duties on alcohol and alcoholic beverages. *OJEU*. 2020;**L256**:1–6. Available from: https://eur‐lex.europa.eu/legal‐content/en/ALL/?uri=CELEX:32020L1151 [145].

• Directive 2010/13/EU of 10 March 2010 on the coordination of certain provisions laid down by law, regulation or administrative action in Member States concerning the provision of audiovisual media services (Audiovisual Media Services Directive). *OJEU*. 2010;**L95**:1–24. Available from: https://eur‐lex.europa.eu/eli/dir/2010/13/oj [132].

• Council Recommendation of 5 June 2001 on the drinking of alcohol by young people, in particular children and adolescents. *OJEU*. 2001;**L161**:38–41. Available from: https://eur‐lex.europa.eu/legal‐content/EN/TXT/?uri=CELEX:32001H0458 [152].

• International Agency for Research on Cancer. Handbook of Cancer Prevention. Alcohol Control Policies. Volume 20B. Lyon: IARC; 2025 [142].

• The SAFER technical package: five areas of intervention at national and subnational levels. Geneva: World Health Organization; 2019. Available from: https://apps.who.int/iris/bitstream/handle/10665/330053/9789241516419‐eng.pdf [150].

• Global alcohol action plan 2022–2030. Geneva: World Health Organization; 2024. Available from: https://iris.who.int/bitstream/handle/10665/376939/9789240090101‐eng.pdf [149].

**Table 6:** Indicators of highest‐level of achievement and European Union (EU) Member States at this level for each of the six MPOWER measures [45].

	Policy areas	Indicator description	EU Member states
**M**	Monitoring of tobacco use and prevention policies	Periodic population‐ and school‐based (13 to 15 years old) surveys with a representative sample of the national population at least every 5 years	Austria, Belgium, Bulgaria, Croatia, Cyprus, Czechia, Denmark, Estonia, Finland, France, Germany, Greece, Hungary, Ireland, Italy, Latvia, Lithuania, Luxembourg, Malta, the Netherlands, Poland, Portugal, Romania, Slovakia, Slovenia, Spain, and Sweden
**P**	Protecting people from tobacco smoke	National smoke‐free laws that ensure all indoor public places are completely smoke‐free (or subnational smoke‐free legislation covering at least 90% of the population)	Bulgaria, Greece, Ireland, Malta, the Netherlands, Romania, and Spain
**O**	Offering help to quit tobacco use	Availability of cost‐covered tobacco cessation support services and NRT prescription and national toll‐free quitlines	Austria, Czechia, Denmark, Ireland, Luxembourg, the Netherlands, Romania, Slovakia and Sweden
**W**	Warning about the dangers of tobacco	Large pictorial warning labels describing specific harmful effects of tobacco use on health are clear, visible and cover at least 50% of tobacco package front and back	Austria, Belgium, Bulgaria, Croatia, Cyprus, Czechia, Denmark, Estonia, Finland, France, Germany, Greece, Hungary, Ireland, Italy, Latvia, Lithuania, Luxembourg, Malta, the Netherlands, Poland, Portugal, Romania, Slovakia, Slovenia, Spain, and Sweden
		National campaigns conducted following a comprehensive communication approach that are aired on television and/or radio	Estonia, France, Ireland, and the Netherlands
**E**	Enforcing bans on TAPS	Complete national ban on all forms of direct and indirect TAPS (or subnational legislation covering at least 90% of the population)	Finland, the Netherlands, Slovenia, and Spain
**R**	Raising taxes on tobacco	Total tobacco taxes share reaching ≥ 75% or over the retail price of the most widely sold brand of manufactured cigarettes	Belgium, Bulgaria, Croatia, Czechia, Denmark, Estonia, Finland, France, Greece, Ireland, Italy, Latvia, Lithuania, Malta, the Netherlands, Poland, Portugal, Slovakia, Slovenia, and Spain

## 4 Conclusions

The European Code Against Cancer, 5th edition (ECAC5), recognises tobacco use, including exposure to SHS, and alcohol consumption, as leading modifiable risk factors for cancer. It also identifies vaping as a strong driver of tobacco smoking among young people who have never smoked. Accordingly, ECAC5 recommends: *‘Do not smoke. Do not use any form of tobacco or vaping products. If you smoke, you should quit’, ‘Keep your home and car free from tobacco smoke’* and *‘Avoid drinking alcohol’* to help individuals reduce their cancer risk. Furthermore, ECAC5 emphasises the importance of reinforcing these individual‐level recommendations with evidence‐based policy actions at the population level to ensure effective cancer prevention across the EU. It provides a set of complementary recommendations for policymakers, grounded in internationally endorsed, cost‐effective policies from authoritative sources.

## Conflict of interest

The authors declare no conflict of interest. Where authors are identified as personnel of the International Agency for Research on Cancer/World Health Organization or the Organisation for Economic Co‐operation and Development, the authors alone are responsible for the views expressed in this article and they do not necessarily represent the decisions, policy or views of the International Agency for Research on Cancer/World Health Organization, or the Organisation for Economic Co‐operation and Development or its member countries. Where authors are affiliated with the European Commission, the contents of this publication do not necessarily reflect the position or opinion of the European Commission.

## Author contributions

AF was responsible for writing the first version of the manuscript. All authors gave critical revisions on the intellectual content of the manuscript and approved the final manuscript.

## Supporting information


**Annex S1.** European Code Against Cancer, 5th edition. © 2026 International Agency for Research on Cancer / WHO. Used with permission.

## Data Availability

The data that supports the findings of this study are available within the figures, tables and/or Supporting Information of this article.
