# Supplementary material for: European Code Against Cancer, 5th edition – tobacco and nicotine containing products, second‐hand smoke, alcohol and cancer
Source: Mol Oncol. 2026 Jan 16;20(1):6–27. doi: 10.1002/1878-0261.70177 (PMC12809474; doi:10.1002/1878-0261.70177)
Supplement: Supplementary file 1 — Annex S1. European Code Against Cancer, 5th edition. © 2026 International Agency for Research on Cancer / WHO. Used with permission. [file MOL2-20-6-s001.pdf]

European Code Against Cancer, 5th edition

14 ways you can help prevent cancer

1 **Smoking**
Do not smoke. Do not use any form of tobacco, or vaping products. If you smoke, you should quit.

2 **Exposure to other people's tobacco smoke**
Keep your home and car free of tobacco smoke.

3 **Overweight and obesity**
Take action to avoid or manage overweight and obesity:

- Limit food high in calories, sugar, fat, and salt.
- Limit drinks high in sugar. Drink mostly water and unsweetened drinks.
- Limit ultra-processed foods.

4 **Physical activity**
Be physically active in everyday life. Limit the time you spend sitting.

5 **Diet**
Eat whole grains, vegetables, legumes, and fruits as a major part of your daily diet. Limit red meat, and avoid processed meat.

6 **Alcohol**
Avoid alcoholic drinks.

7 **Breastfeeding**
Breastfeed your baby for as long as possible.

8 **Sun exposure**
Avoid too much sun exposure, especially for children. Use sun protection. Never use sunbeds.

9 **Cancer-causing factors at work**
Inform yourself about cancer-causing factors at work, and call on your employer to protect you against them. Always follow health and safety instructions at your workplace.

10 **Indoor radon gas**
Inform yourself about radon gas levels in your area by checking a local radon map. Seek professional help to measure levels in your home and, if necessary, reduce them.

11 **Air pollution**
Take action to reduce exposure to air pollution by:

- Using public transportation, and walking or cycling instead of using a car
- Choosing low-traffic routes when walking, cycling, or exercising
- Keeping your home free of smoke by not burning materials such as coal or wood
- Supporting policies that improve air quality.

12 **Cancer-causing infections**

- Vaccinate girls and boys against hepatitis B virus and human papillomavirus (HPV) at the age recommended in your country.
- Take part in testing and treatment for hepatitis B and C viruses, human immunodeficiency virus (HIV), and *Helicobacter pylori,* as recommended in your country.

13 **Hormone replacement therapy**
If you decide to use hormone replacement therapy (for menopausal symptoms) after a thorough discussion with your health-care professional, limit its use to the shortest duration possible.

14 **Organized cancer screening programmes**
Take part in organized cancer screening programmes, as recommended in your country, for:

- Bowel cancer
- Breast cancer
- Cervical cancer
- Lung cancer.

To find out more, please visit:

International Agency for Research on Cancer
World Health Organization
Co-funded by the European Union

Recommendations for policy-makers on implementing the European Code Against Cancer, 5th edition

A Cancer Plan for Europe

An estimated 1.3 million lives were lost due to cancer in the European Union (EU) in 2022. New cancer diagnoses are projected to increase by about 18% and cancer deaths by 26% by 2040.[1] Recent estimates indicate that the overall costs of cancer in the EU amount to more than **€93 billion annually**.[2] Beyond ethical imperatives, there are strong economic motives to effectively control cancer.

Acknowledging the societal challenge presented by the rising cancer burden in the EU, in 2021 the European Commission (EC) launched **Europe's Beating Cancer Plan** to mobilize resources to support Member States in their efforts to control cancer. The Cancer Plan is grounded in prevention, based on current scientific knowledge that about **40% of cancer cases are preventable**.[3] Ambitious targets have been set for the implementation of the Europe's Beating Cancer Plan, but these targets will continue to remain out of reach without collaborative efforts from all of society. This is where the European Code Against Cancer has a pivotal role.

What is the European Code Against Cancer (ECAC) and its purpose?

A long-standing initiative of the EC, the ECAC proposes a series of actions and interventions anyone can take to help prevent cancer. The latest, 5th edition of the ECAC (ECAC5) consists of **14 recommendations** based on current scientific evidence on **personal behavioural factors, environmental factors, and medical interventions**, specific to the general population in the EU. For the first time, ECAC5 is aimed not only at individuals but also at policy-makers, including 14 complementary recommendations on **population-level measures** that may reinforce the recommendations for individuals. Individuals are encouraged to take proactive steps to live healthier lives, as outlined in the recommendations. Policy-makers are provided with guidance, which considers structural factors and the health systems context, to implement policies based on science **to enable environments** where everyone can make informed healthy choices and adopt the recommendations.

These recommendations provide co-benefits to prevent other noncommunicable diseases (NCDs) with similar underlying risk factors, and opportunities for health promotion. Together, the ECAC5 recommendations provide a roadmap to reduce cancer risk, tackle misconceptions about cancer, and improve public health and well-being.

Co-benefits for other NCDs with similar risk factors and opportunities for health promotion

Personal behavioural factors

Recommendations #1 to #6

Adopting healthy behaviours such as not smoking, avoiding alcohol, maintaining a balanced diet, and engaging in regular physical activity not only reduces cancer risk but also lowers the incidence of heart disease, diabetes, and respiratory conditions.

Recommendation #7

Breastfeeding benefits both mother and child. For mothers, it supports post-partum weight loss and reduces obesity-related health risks later in life. For children, it aids nutrition, development, and infection prevention.

Recommendation #8

Excessive exposure to ultraviolet (UV) radiation may alter immune responses, which could potentially affect autoimmune disorders.

Environmental factors

Recommendations #9 to #11

Mitigating exposures in the workplace and daily environment can also contribute to reducing the risk for respiratory diseases.

Medical interventions

Recommendation #12

Comprehensive prevention and treatment strategies for infection-related cancer also prevent severe diseases related to or caused by these infections, such as liver cirrhosis.

Recommendation #13

Hormone replacement therapy (HRT) does not protect the heart or brain. On the contrary, HRT appears to increase the risk of dementia.

Recommendation #14

Participating in cancer screening may be an opportunity to learn about healthy behaviours that can reduce the risk of cancer and other NCDs

For whom are the policy recommendations?

The 14 policy recommendations are primarily aimed at policy-makers, as individuals with power to influence or determine policies and practices at an international, national, regional, or local level. These policy recommendations can also be used by civil society to advocate for policy changes towards cancer prevention. Health professionals are also a key audience, because they play a vital role in advocating for and implementing these recommendations.

How were the policy recommendations developed?

The recommendations for policy-makers are underpinned by **established authoritative policies** produced by the EU, the World Health Organization (WHO), and other international bodies, which are detailed in the references provided. The relevance and suitability of these policies was assessed using a **hierarchization of authoritative sources and prioritization strategy** based on the Nuffield Ladder of Interventions[4] or the Hierarchy of Prevention and Control Measures.[5] The result is a summary of the most relevant existing international policies, protected from vested interests, that may support each of the recommendations for the public and for which they may demand action. In most cases, the recommendations are endorsed by **Europe's Beating Cancer Plan**[6] and the **WHO best buys for NCDs**.[7]

Which key principles should guide the implementation of ECAC5?

Policy-makers are urged to:

- adopt the recommendations, in a **phased implementation** manner when recommendations require infrastructure that is not available when ECAC5 is published
- make the **healthy choice the easiest** – most affordable, accessible, and available – option in all settings
- ensure that all recommendations are implemented with an **equity perspective** by addressing vulnerable populations, including people experiencing socioeconomic disadvantage
- **safeguard the integrity** of all recommendations by taking appropriate steps to protect against undue industry interference
- invest in regular **capacity-building**, and **monitoring and evaluation**.

For effective dissemination and implementation, it is recommended that ECAC5 is promoted as a whole, without changes to the text. It is strongly advised not to edit, delete, or substitute words that may affect the meaning of the recommendations, because they have been deliberately chosen and carefully translated into 23 EU languages to maintain, as far as possible, the original meaning.

Policy-makers are also urged to support and engage in efforts to actively communicate the ECAC5 recommendations.

References

1. Ferlay J, Laversanne M, Ervik M, Lam F, Colombet M, Mery L, Piñeros M, Znaor A, Soerjomataram I, Bray F (2024). Global Cancer Observatory: Cancer Tomorrow (version 1.1). Lyon, France: International Agency for Research on Cancer. Available from: https://gco.iarc.who.int/tomorrow
2. OECD (2024), Tackling the Impact of Cancer on Health, the Economy and Society, OECD Health Policy Studies, OECD Publishing, Paris. Available from: https://doi.org/10.1787/85e7c3ba-en
3. GBD 2019 Cancer Risk Factors Collaborators. The global burden of cancer attributable to risk factors, 2010–19: a systematic analysis for the Global Burden of Disease Study 2019. Lancet. 2022;400(10352):563–91 doi:10.1016/S0140-6736(22)01438-6
4. Nuffield Council on Bioethics. Public health: ethical issues. London: Nuffield Council on Bioethics; 2007. Available from: https://www.nuffieldbioethics.org/publication/public-health-ethical-issues/
5. European Agency for Safety and Health at Work. Hierarchy of prevention and control measures. OSHWiki. Available from: https://oshwiki.osha.europa.eu/en/themes/hierarchy-prevention-and-control-measures
6. European Commission. Communication from the Commission to the European Parliament and the Council: Europe's Beating Cancer Plan. COM/2021/44 final. Brussels: European Commission; 2021. Available from: https://eur-lex.europa.eu/legal-content/EN/TXT/?uri=COM:2021:44:FIN
7. Tackling NCDs: best buys and other recommended interventions for the prevention and control of noncommunicable diseases, second edition. Geneva: World Health Organization; 2024. Available from: https://www.who.int/publications/i/item/9789240091078.

1. Tobacco and nicotine-containing products

- Adopt, implement, and enforce comprehensive tobacco control policies, as per the WHO Framework Convention on Tobacco Control, including:
  - Measures to raise tobacco taxes to at least 75% of tobacco's retail price and significantly increase tobacco taxes every year. All tobacco products should be taxed in a comparable way as appropriate, in particular where the risk of substitution exists.
  - Measures to restrict the availability and accessibility of tobacco products. This includes increasing the age of sale and allowing the sale of tobacco products only in licensed stores.
  - Measures to ban tobacco advertising, promotion, and sponsorship, including display bans at the point of sale.
  - Provision of smoking cessation services. Identify and allocate sustainable funding for tobacco cessation and tobacco dependence treatment programmes.
  - Large graphic health warnings, labelling, and plain, standardized packaging for tobacco products.
- Extend such regulations to apply to all tobacco products, electronic cigarettes, and all novel tobacco and nicotine-containing products.
- Establish and work towards achieving a goal for a tobacco-free generation in your country.
- Complementing the above-mentioned policy measures, implement regular public health campaigns to raise awareness of the damaging effects of tobacco and the benefits of smoking cessation.

References

- World Health Organization Framework Convention on Tobacco Control. Geneva: WHO; 2003. Available from: https://apps.who.int/iris/rest/bitstreams/50793/retrieve
- Directive 2014/40/EU of 3 April 2014 on the approximation of the laws, regulations and administrative provisions of the Member States concerning the manufacture, presentation and sale of tobacco and related products and repealing Directive 2001/37/EC. *Official Journal of the European Union*. 2014 Apr 29;L127:1–38. Available from: https://eur-lex.europa.eu/eli/dir/2014/40/oj
- Council Directive (EU) 2020/262 of 19 December 2019 laying down the general arrangements for excise duty (recast). *Official Journal of the European Union*. 2020 Feb 27;L58:4–21. Available from: https://eur-lex.europa.eu/eli/dir/2020/262/oj
- Directive 2010/13/EU of 10 March 2010 on the coordination of certain provisions laid down by law, regulation or administrative action in Member States concerning the provision of audiovisual media services (Audiovisual Media Services Directive). *Official Journal of the European Union*. 2010 Apr 15;L95:1–24. Available from: https://eur-lex.europa.eu/eli/dir/2010/13/oj

2. Second-hand smoke

- Enforce legislation to eliminate exposure to second-hand tobacco smoke in all indoor workplaces, public places, and transportation.
- Extend smoke-free laws to outdoor public places, in particular health-care centres and areas where children and adolescents could be exposed, such as educational settings and playgrounds.
- Extend smoke-free legislation to include all novel tobacco and nicotine-containing products.
- Complementing the above-mentioned policy measures, implement regular smoke-free environment campaigns for private settings, such as homes and vehicles, and regular public health campaigns to raise awareness of the effects of exposure to second-hand smoke on health and the risk of cancer.

References

- World Health Organization Framework Convention on Tobacco Control. Geneva: WHO; 2003. Available from: https://apps.who.int/iris/rest/bitstreams/50793/retrieve
- Council Recommendation of 3 December 2024 on smoke- and aerosol-free environments replacing Council Recommendation 2009/C 296/02. *Official Journal of the European Union*. 2024 Dec 3;C7425:1–5. Available from: https://eur-lex.europa.eu/eli/C/2024/7425

3. Overweight and obesity

- Implement fiscal policies:
  - Increase taxes and prices of foods high in sugars, fat, or salt, including processed meat, and sugar-sweetened beverages.
  - Lower taxes and prices of whole-grain products, vegetables, legumes, and fruit.

- Make the healthy choice the easiest – most affordable, accessible, and available – option in all settings:
    - Increase the availability, visibility, and affordability of whole-grain products, vegetables, legumes, and fruit.
    - Remove snacks high in sugars, fat, or salt and sweetened beverages from vending machines and other locations. Make the sugar-free option the default option for hot beverages in vending machines.
    - Provide fresh filtered tap water in all settings.
- Implement procurement policies with mandatory standards that limit foods high in sugars, fat, or salt, including processed meat, and sugar-sweetened beverages in all settings. Specify upper limits for total daily energy from sugars and an upper limit of sodium intake per meal.
- Ban or restrict marketing, advertising, and promotion of foods high in sugars, fat, or salt, especially to children.
- Introduce and promote weight management interventions that are accessible and affordable for all citizens.
- Agree upon and implement an effective EU-wide front-of-pack nutrition labelling scheme that is understood by all consumers.
- Update and promote national food-based dietary guidelines.
- Update curricula to include nutrition education classes across the EU.
- Complementing the above-mentioned policy measures, implement regular public health campaigns to raise awareness of the association of excess body weight with cancer risk and the importance of prevention of overweight and obesity.

References

- Directive 2010/13/EU of 10 March 2010 on the coordination of certain provisions laid down by law, regulation or administrative action in Member States concerning the provision of audiovisual media services (Audiovisual Media Services Directive). *Official Journal of the European Union*. 2010 Apr 15;L95:1–24. Available from: https://eur-lex.europa.eu/eli/dir/2010/13/oj
- European Union Action Plan on Childhood Obesity 2014–2020. Brussels: European Commission; 2014. Available from: https://health.ec.europa.eu/publications/eu-action-plan-childhood-obesity-2014-2020
- Commission Delegated Regulation (EU) 2017/40 of 3 November 2016 supplementing Regulation (EU) No 1308/2013 of the European Parliament and of the Council with regard to the Union aid scheme for the supply of fruit and vegetables, bananas and milk in educational establishments and amending Commission Implementing Regulation (EU) No 907/2014. *Official Journal of the European Union*. 2017 Jan 10;L5:11–22. Available from: https://eur-lex.europa.eu/eli/reg_del/2017/40/oj
- Food-Based Dietary Guidelines in Europe. Brussels: European Commission. Available from: https://knowledge4policy.ec.europa.eu/health-promotion-knowledge-gateway/topic/food-based-dietary-guidelines-europe
- Global report on the use of sugar-sweetened beverage taxes. Geneva: World Health Organization; 2023. Available from: https://www.who.int/publications/i/item/9789240084995

4. Physical activity

- Implement fiscal incentives for all forms of active travel.
- Promote and enable active public transportation for all ages and abilities, including vulnerable groups, by investing in suitable infrastructure.
- Enhance urban planning policies to create safer, greener environments that encourage walking, cycling, and other mobility options in both urban and rural areas; strengthen the policy and design guidelines for public amenities, workplaces, and social housing to enable citizens with diverse abilities to have access and be physically active in and around buildings.
- Introduce or reinforce physical education classes across the EU, with both curricula and infrastructure that allow for maximum inclusiveness.
- Promote physical activity at work with initiatives and infrastructure standards.
- Implement incentives for employers to provide opportunities for physical activity.
- Introduce physical activity on prescription in primary care as a tool for prevention of noncommunicable diseases.
- Work with vulnerable groups to address barriers to engaging in physical activity.

- Update and promote EU-wide and national physical activity guidelines.
- Complementing the above-mentioned policy measures, implement regular public health campaigns to raise awareness of the benefits of physical activity in the prevention of cancer.

References

- Council Recommendation of 26 November 2013 on promoting health-enhancing physical activity across sectors. *Official Journal of the European Union*. 2013 Dec 4;C354:1–5. Available from: https://eur-lex.europa.eu/legal-content/GA/TXT/?uri=celex:32013H1204(01)
- European Union Physical Activity Guidelines: Recommended policy actions in support of health-enhancing physical activity. Brussels: European Commission; 2008. Available from: https://health.ec.europa.eu/document/download/97d6f94c-f4bd-4530-a488-c5f97f969a80_en?filename=2008_eu_physical_activity_guidelines_en.pdf
- European Union Action Plan on Childhood Obesity 2014–2020. Brussels: European Commission; 2014. Available from: https://health.ec.europa.eu/publications/eu-action-plan-childhood-obesity-2014-2020
- Global action plan on physical activity 2018–2030: more active people for a healthier world. Geneva: World Health Organization; 2018. Available from: https://www.who.int/publications/i/item/9789241514187

5. Diet

- Implement fiscal policies:
  - Increase taxes and prices of processed meat.
  - Lower taxes and prices of whole-grain products, vegetables, and fruit.
- Make the healthy choice the easiest – most affordable, accessible, and available – option in all settings:
  - Implement procurement policies with mandatory standards that limit red and processed meat in all settings.
  - Increase the availability, visibility, and affordability of whole-grain products, vegetables, legumes, and fruit.
  - Set mandatory standards that limit or ban foods high in sugars, fat, or salt.
- Agree upon and implement an effective EU-wide front-of-pack nutrition labelling scheme that is understood by all consumers.
- Update and promote national food-based dietary guidelines.
- Update curricula to include nutrition education classes across the EU.
- Complementing the above-mentioned policy measures, implement regular public health campaigns to raise awareness of the importance of healthy nutrition in the prevention of cancer.

References

- European Union Action Plan on Childhood Obesity 2014–2020. Brussels: European Commission; 2014. Available from: https://health.ec.europa.eu/publications/eu-action-plan-childhood-obesity-2014-2020
- Commission Delegated Regulation (EU) 2017/40 of 3 November 2016 supplementing Regulation (EU) No 1308/2013 of the European Parliament and of the Council with regard to the Union aid scheme for the supply of fruit and vegetables, bananas and milk in educational establishments and amending Commission Implementing Regulation (EU) No 907/2014. *Official Journal of the European Union*. 2017 Jan 10;L5:11–22. Available from: https://eur-lex.europa.eu/eli/reg_del/2017/40/oj
- Food-Based Dietary Guidelines in Europe. Brussels: European Commission. Available from: https://knowledge4policy.ec.europa.eu/health-promotion-knowledge-gateway/topic/food-based-dietary-guidelines-europe

6. Alcohol

- Increase prices of alcohol through taxation to make alcohol less affordable.
- Establish a minimum price for all alcoholic beverages.
- Restrict the availability and accessibility of all alcoholic beverages.
- Ensure that no alcoholic beverages are offered in any public catering services.
- Increase minimum legal age limits to at least 19 years for selling and serving all alcoholic beverages.
- Ban or restrict advertising, promotion, and sponsorship of all alcoholic beverages in all media and for all purposes, especially those targeting minors.
- Facilitate access to screening, brief interventions, and treatment of alcohol use disorder in primary care and community settings.

- Introduce health warning labels related to alcohol consumption and nutrition labelling on all alcoholic beverages.
- Complementing the above-mentioned policy measures, implement regular public health campaigns to raise awareness of the detrimental effects of alcohol intake on health and its association with cancer risk.

References

- Council Directive (EU) 2020/262 of 19 December 2019 laying down the general arrangements for excise duty (recast). *Official Journal of the European Union*. 2020 Feb 27;L58:4–21. Available from: https://eur-lex.europa.eu/eli/dir/2020/262/oj
- Council Directive (EU) 2020/1151 of 29 July 2020 amending Directive 92/83/EEC on the harmonization of the structures of excise duties on alcohol and alcoholic beverages. *Official Journal of the European Union*. 2020 Aug 5;L256:1–6. Available from: https://eur-lex.europa.eu/legal-content/en/ALL/?uri=CELEX:32020L1151
- Directive 2010/13/EU of 10 March 2010 on the coordination of certain provisions laid down by law, regulation or administrative action in Member States concerning the provision of audiovisual media services (Audiovisual Media Services Directive). *Official Journal of the European Union*. 2010 Apr 15;L95:1–24. Available from: https://eur-lex.europa.eu/eli/dir/2010/13/oj
- Council Recommendation of 5 June 2001 on the drinking of alcohol by young people, in particular children and adolescents. *Official Journal of the European Communities*. 2001 Jun 16;L161:38–41. Available from: https://eur-lex.europa.eu/legal-content/EN/TXT/?uri=CELEX:32001H0458
- IARC (2025). Alcohol Policies. IARC Handb Cancer Prev. 20B: 1-282. Available from: https://publications.iarc.who.int/Book-And-Report-Series/Iarc-Handbooks-Of-Cancer-Prevention/Alcohol-Policies-2025
- The SAFER technical package: five areas of intervention at national and subnational levels. Geneva: World Health Organization; 2019. Available from: https://apps.who.int/iris/bitstream/handle/10665/330053/9789241516419-eng.pdf
- Global alcohol action plan 2022–2030. Geneva: World Health Organization; 2024. Available from: https://iris.who.int/bitstream/handle/10665/376939/9789240090101-eng.pdf

7. Breastfeeding

- Ensure compliance with the International Code of Marketing of Breast-Milk Substitutes, adopting and enforcing regulation to protect breastfeeding from inappropriate marketing of food products that compete with breastfeeding. Breast-milk substitutes should be available when needed but should not be promoted.
- Establish and enforce policies that ensure a sufficient duration of parental leave, as well as flexible working arrangements to enable working mothers to exclusively breastfeed their infants for six months and to continue thereafter.
- Enact policies and introduce incentives for employers to provide breastfeeding-friendly environments.
- Encourage breastfeeding-friendly policies and facilities in public areas, and protect the right of women to breastfeed whenever and wherever they need to.
- Establish breastfeeding support networks. Train health-care professionals to support new mothers in breastfeeding, and make breastfeeding consultations accessible for all mothers.
- Complementing the above-mentioned policy measures, implement regular public health campaigns to raise awareness of breastfeeding and its health benefits for both the baby and the mother.

References

- International Code of Marketing of Breast-Milk Substitutes. Geneva: World Health Organization; 1981. Available from: https://www.who.int/publications/i/item/9241541601
- Implementation guidance: protecting, promoting, and supporting breastfeeding in facilities providing maternity and newborn services: the revised Baby-friendly Hospital Initiative 2018. Geneva: World Health Organization; 2018. Available from: https://www.who.int/publications/i/item/9789241513807
- Guidance on regulatory measures aimed at restricting digital marketing of breast-milk substitutes. Geneva: World Health Organization; 2023. Available from: https://www.who.int/publications/i/item/9789240084490
- Protection, promotion and support of breastfeeding in Europe: a blueprint for action. European Commission, Directorate Public Health and Risk Assessment, Luxembourg, 2004. Available from: http://europa.eu.int/comm/health/ph_projects/2002/promotion/promotion_2002_18_en.htm

8. Sun and ultraviolet (UV) radiation exposure

- Harmonize and enforce policies and recommendations on protection from exposure to UV radiation across the EU.
- Continue to support measures to reduce exposure to UV radiation in the public and especially in children, including from sunbeds and excess solar UV radiation.

- Provide collective protection from sun exposure, such as shading infrastructures and greening, at the local level.
- In the workplace, provide organizational measures, shading, and access to UV-safe clothing or other collective and individual protective equipment to reduce exposure of workers to solar and artificial UV radiation.
- Complementing the above-mentioned policy measures, invest in and promote regular public health campaigns to raise awareness and knowledge of exposure to UV radiation and cancer risk, and monitor their effectiveness in changing behaviour and reducing exposure.

References

- Directive 2006/25/EC of 5 April 2006 on the minimum health and safety requirements regarding the exposure of workers to risks arising from physical agents (artificial optical radiation). *Official Journal of the European Union*. 2006 Apr 27;L114:38–59. Available from: https://eur-lex.europa.eu/legal-content/EN/TXT/?uri=CELEX:32006L0025
- Directive 89/391/EEC of 12 June 1989 on the introduction of measures to encourage improvements in the safety and health of workers at work (OSH "Framework Directive"). *Official Journal of the European Union*. 1989 Jun 29;L183:1–8. Available from: https://eur-lex.europa.eu/legal-content/EN/TXT/?uri=CELEX:01989L0391-20081211
- SCHEER (Scientific Committee on Health, Environmental and Emerging Risks), Opinion on Biological effects of ultraviolet radiation relevant to health with particular reference to sunbeds for cosmetic purposes, 2016. Available from: https://ec.europa.eu/health/scientific_committees/scheer/docs/scheer_o_003.pdf

9. Cancer-causing factors at work

- Scale up efforts to enforce existing EU legislation on occupational carcinogens, including compliance with binding occupational exposure limits.
- Encourage all economic sectors with exposure to carcinogens to work with social partners to develop and implement social dialogue agreements for reduction of the prevalence and levels of exposure, and to monitor and publish key performance indicators. Support small and medium-sized enterprises and self-employed workers to actively engage with such initiatives.
- Include specific occupational safety and health (OSH) requirements in the criteria for public procurement, to support the elimination and/or reduction of workers' exposure to carcinogens in the workplace.
- Ensure that knowledge on safe work practices and how to prevent exposure to carcinogens is integrated into education programmes, including in vocational training.

References

- Directive 89/391/EEC of 12 June 1989 on the introduction of measures to encourage improvements in the safety and health of workers at work (OSH "Framework Directive"). *Official Journal of the European Union*. 1989 Jun 29;L183:1–8. Available from: https://eur-lex.europa.eu/legal-content/EN/TXT/?uri=CELEX:01989L0391-20081211
- Directive 2004/37/EC of 29 April 2004 on the protection of workers from the risks related to exposure to carcinogens, mutagens or reprotoxic substances at work. *Official Journal of the European Union*. 2004 Jun 30;L229:23–34. Available from: https://eur-lex.europa.eu/eli/dir/2004/37
- Directive 2009/148/EC of 30 November 2009 on the protection of workers from the risks related to exposure to asbestos at work. *Official Journal of the European Union*. 2009 Dec 16;L330:28–36. Available from: https://eur-lex.europa.eu/eli/dir/2009/148
- Regulation (EU) 2023/1542 of 12 July 2023 concerning batteries and waste batteries. *Official Journal of the European Union*. 2023 Jul 18;L191:1–102. Available from: https://eur-lex.europa.eu/eli/reg/2023/1542
- Roadmap on Carcinogens: A European-wide voluntary action scheme to tackle work-related cancer [internet]. [accessed 28 January 2025]. Available from: https://stopcarcinogensatwork.eu

10. Indoor radon gas

- Enforce basic safety standards for the protection of individuals' health against radon exposure. Adapt the existing EU Directive on ionizing radiation to include alpha radiation emitters such as radon as a source of ionizing radiation in building materials.
- Develop general awareness programmes for radon, make user-friendly tools available that include radon prediction maps at the residential, school, and workplace level, and increase population-based radon testing.
- Provide financial support for radon remediation in homes and other buildings.
- Invest in training of recognized public and private bodies for workplace and residential radiation protection.

References

- Directive 2013/59/EURATOM of 5 December 2013 laying down basic safety standards for protection against the dangers arising from exposure to ionising radiation. *Official Journal of the European Union*. 2014 Jan 17;L13:1–73. Available from: https://eur-lex.europa.eu/eli/dir/2013/59
- Protection against exposure due to radon indoors and gamma radiation from construction materials — Methods of prevention and mitigation, IAEA-TECDOC-1951. Vienna: International Atomic Energy Agency; 2021. Available from: https://www-pub.iaea.org/MTCD/publications/PDF/TE-1951web.pdf

11. Air pollution

- Fully align EU air quality limit values with WHO global air quality guidelines for outdoor air pollution without delay. At the local and national levels, develop and implement plans to ensure that levels of all air pollutants comply with the WHO guidelines.
- Ensure further reductions in industrial emissions.
- Align policies limiting air pollution with climate change, energy, and other environmental policies at the EU, national, and local levels to capitalize on co-benefits. Policies should be targeted at different levels of governance.
- Improve spatial planning to reduce motorized traffic and provide accessible and safe infrastructure for active and greener travel.
- Develop and implement policies to discourage and phase out outdoor and indoor fossil and solid fuels for heating, cooking, and recreational purposes, accompanied by awareness-raising campaigns.
- Incentivize cleaner forms of energy for heating and cooking, which do not adversely affect indoor and outdoor air quality, such as heat pumps, solar power, or geothermal energy.
- Support citizens to actively engage and participate in developing local plans to reduce emissions of air pollutants. Make sure information on outdoor air pollution at the local and national levels is easily available for the public.
- Protect sensitive populations and vulnerable groups from air pollution; for example, do not locate new schools or nursing homes next to busy roads. Where existing schools and other buildings with sensitive populations and vulnerable groups are situated next to busy roads, incentivize the use and correct maintenance of air filters and purifiers to avoid or decrease infiltration of outdoor air pollution.

References

- Directive (EU) 2024/2881 of 23 October 2024 on ambient air quality and cleaner air for Europe (recast). *Official Journal of the European Union*. 2024 Oct 23;L374:1–45. Available from: https://eur-lex.europa.eu/eli/dir/2024/2881
- Directive 2010/75/EU of 24 November 2010 on industrial emissions (integrated pollution prevention and control). *Official Journal of the European Union*. 2010 Dec 17;L334:17–119. Available from: https://eur-lex.europa.eu/eli/dir/2010/75
- European Union Action Plan: Towards Zero Pollution for Air, Water and Soil. Brussels: European Commission; 2021. Available from: https://eur-lex.europa.eu/legal-content/EN/TXT/?uri=CELEX:52021DC0400
- Measures to reduce emissions of air pollutants and greenhouse gases: the potential for synergies. Copenhagen: European Environment Agency (EEA); 2022. Available from: https://www.eea.europa.eu/publications/measures-to-reduce-emissions-of
- Global air quality guidelines: particulate matter (PM2.5 and PM10), ozone, nitrogen dioxide, sulphur dioxide and carbon monoxide. Geneva: World Health Organization; 2021. Available from: https://iris.who.int/handle/10665/345329
- Guidelines for indoor air quality: household fuel combustion. Geneva: World Health Organization; 2014. Available from: https://apps.who.int/iris/bitstream/handle/10665/141496/9789241548885_eng.pdf
- Personal-level actions to reduce air pollution exposure in the WHO European Region. Copenhagen: WHO Regional Office for Europe; 2024. Available from: https://iris.who.int/bitstream/handle/10665/375889/WHO-EURO-2024-9115-48887-72806-eng.pdf?sequence=1

12. Cancer-causing infections and related interventions

- Strengthen hepatitis B virus (HBV) vaccination programmes to maximize their effect on reducing the prevalence of HBV infection. This can be achieved, according to the epidemiological burden, by ensuring that:
  - All children receive their first dose of HBV vaccine as soon as possible after birth.
  - HBV vaccination programmes are resourced to reach the 95% coverage target.
  - Catch-up vaccinations are offered to people at increased risk of acquiring HBV infection.
- Strengthen human papillomavirus (HPV) vaccination programmes to maximize their impact by ensuring that:

    - The vaccine is given at the youngest age possible to the priority target age group (between 9–14 years) as decided at the national level.
    - HPV vaccination programmes are resourced to reach the 90% coverage target for girls and boys.
    - Catch-up vaccination opportunities are provided to people older than the priority target age but at least until age 18 years, when feasible.
    - Individuals at high risk of HPV infection, including immunocompromised individuals and people who experienced sexual abuse, are considered for vaccination against HPV as a priority. Individuals known to be immunocompromised or infected with human immunodeficiency virus (HIV) should receive at least two HPV vaccine doses and, where possible, three doses.
- Strengthen the importance of HBV and HPV vaccination as cancer prevention tools. This includes identifying behavioural determinants of vaccine uptake, addressing obstacles to vaccination, and implementing awareness-raising campaigns to increase confidence in these vaccines among health professionals, teachers, parents, and (pre-)adolescents. Monitor progress in vaccination programmes against HBV and HPV in a timely manner.
- Introduce sustainable initiatives of testing and treating:
    - Adopt policies facilitating the offer of an affordable, ideally free of charge, test for HBV, hepatitis C virus (HCV), HIV, and *Helicobacter pylori* (*H. pylori*) to adults in low-threshold settings using a non-stigmatizing approach.
    - Treat individuals with confirmed HCV, HIV, or *H. pylori* infection as early as possible. For HBV, treatment should be provided to selected individuals, according to the clinical guidelines.
    - Offer pregnant women HBV and HIV testing, and consider offering also HCV testing based on individual risk assessment.
    - For HIV, the offer of testing should prioritize individuals with HIV indicator conditions and people at increased risk of sexual acquisition of HIV or exposure to blood and blood products.
    - Develop and coordinate public health awareness campaigns related to all infections that cause cancer and interventions that avoid their acquisition or progression to disease.
    - Monitor progress in test-and-treat strategies in the population, including low-literacy and vulnerable groups.

References

- Council Recommendation of 21 June 2024 on vaccine-preventable cancers. *Official Journal of the European Union*. 2024 Jun 28;C4259:1–8. Available from: https://eur-lex.europa.eu/eli/C/2024/4259
- Council recommendation on strengthening prevention through early detection: a new EU approach on cancer screening replacing Council Recommendation 2003/878/EC. Brussels: European Commission; 2022. Available from: https://eur-lex.europa.eu/legal-content/EN/TXT/?uri=celex:32022H1213(01)
- Prevention of hepatitis B and C in the EU/EEA. Stockholm: European Centre for Disease Prevention and Control (ECDC); 2022. Available from: https://www.ecdc.europa.eu/assets/Prevention-Hepatitis-B-and-C/index.html
- Public health guidance on HIV, hepatitis B and C testing in the EU/EEA: an integrated approach. Stockholm: European Centre for Disease Prevention and Control (ECDC); 2018. Available from: https://www.ecdc.europa.eu/sites/default/files/documents/hiv-hep-testing-guidance_0.pdf
- Regional action plans for ending AIDS and the epidemics of viral hepatitis and sexually transmitted infections 2022–2030. Copenhagen: WHO Regional Office for Europe; 2023. Available from: https://iris.who.int/bitstream/handle/10665/369243/9789289058957-eng.pdf?sequence=7
- Guidelines for the prevention, diagnosis, care and treatment for people with chronic hepatitis B infection. Geneva: World Health Organization; 2024. Available from: https://iris.who.int/bitstream/handle/10665/376353/9789240090903-eng.pdf?sequence=1
- Human papillomavirus vaccines: WHO position paper (2022 update). Geneva: World Health Organization; 2022. Available from: https://www.who.int/publications/i/item/who-wer9750-645-672
- Park JY, editor Population-based Helicobacter pylori screen-and-treat strategies for gastric cancer prevention: guidance on implementation (IARC Working Group Reports No. 12). Lyon, France: International Agency for Research on Cancer; 2025. Available from: https://publications.iarc.who.int/648. Licence: CC BY-NC-ND 3.0 IGO.

13. Hormone replacement therapy

- Make provisions for:
    - Easy access to health-care professionals for women to discuss their menopausal symptoms and the benefits and harms of using hormone replacement therapy (HRT) and non-hormonal alternatives.

- Assessment of baseline cancer risk, including mammography before starting to use HRT, where applicable.
- Availability, on a prescription-only basis, of various formulations to personalize use of HRT and minimize risks.
- Periodic re-evaluation of symptoms and HRT use.

References

- Menopause: identification and management. National Institute for Health and Care Excellence (NICE) guideline NG23. London: NICE; 2024. Available from: https://www.nice.org.uk/guidance/ng23

14. Organized cancer screening programmes

- Implement sustainable, organized screening programmes for colorectal (bowel), breast, and cervical cancer:*
  - For colorectal cancer screening, implement quantitative faecal immunochemical test (FIT) every two years for individuals aged 50–74 years. Once-only endoscopy may be considered as an alternative strategy within the same age range.
  - For breast cancer screening, implement digital mammography every two years for women** aged 50–69 years, and consider implementing it for women aged 45–49 years and 70–74 years. Other screening tools or additional examinations should be considered for women with high mammographic density.
  - For cervical cancer screening, implement HPV screening at intervals no shorter than five years for women** aged 30–65 years. Policies can be adapted according to vaccination status and screening history.
- Implement sustainable, organized screening programmes for lung cancer.* Implement low-dose computed tomography every year (preferred) or every two years with integrated smoking cessation interventions for individuals identified as being at increased risk of lung cancer based on criteria of either age and history of smoking or locally validated multivariable risk models.

** The recommendations are subject to updates to reflect scientific and technological advances as specified in the European Guidelines for Cancer Screening and Diagnosis: https://cancer-screening-and-care.jrc.ec.europa.eu*

*** Includes people assigned female at birth who are eligible for this screening.*

References

- Council recommendation on strengthening prevention through early detection: a new EU approach on cancer screening replacing Council Recommendation 2003/878/EC. Brussels: European Commission; 2022. Available from: https://eur-lex.europa.eu/legal-content/EN/TXT/?uri=celex:32022H1213(01)
- European Commission: Directorate-General for Health and Consumers, Executive Agency for Health and Consumers, World Health Organization, Karsa L, Patnick J, Segnan N. *European guidelines for quality assurance in colorectal cancer screening and diagnosis*. Publications Office; 2010. Available from: doi:10.2772/1458
- International Agency for Research on Cancer. Handbook of Cancer Prevention. Colorectal cancer screening. Volume 17. Lyon: IARC; 2019. Available from: http://publications.iarc.fr/573
- European Commission Initiative on Breast Cancer (ECIBC). European guidelines on breast cancer screening and diagnosis. Available from: https://cancer-screening-and-care.jrc.ec.europa.eu/en/ecibc/european-breast-cancer-guidelines
- European Commission: Directorate-General for Health and Food Safety, Karsa L, Dillner J, Suonio E, Törnberg S, Anttila A, Ronco G et al. *European guidelines for quality assurance in cervical cancer screening : second edition : supplements*. Publications Office; 2015. Available from: doi:10.2875/859507
- WHO guideline for screening and treatment of cervical pre-cancer lesions for cervical cancer prevention, second edition. Geneva: World Health Organization; 2021. Available from: https://www.who.int/publications/i/item/9789240030824
- Baldwin DR, O'Dowd EL, Tietzova I, et al. Developing a pan-European technical standard for a comprehensive high-quality lung cancer computed tomography screening programme: an ERS technical standard. *Eur Respir J*. 2023;61(3):2300128.doi:10.1183/13993003.00128-2023
- O'Dowd EL, Tietzova I, Bartlett E, et al. ERS/ESTS/ESTRO/ESR/ESTI/EFOMP statement on management of incidental findings from low dose CT screening for lung cancer. *Eur Respir J*. 2023;61(6):2300533. doi:10.1183/13993003.00533-2023

Co-funded by the
European Union

Funded by the European Union. Views and opinions expressed are however those of the authors only and do not necessarily reflect those of the European Union or European Health and Digital Executive Agency (HaDEA). Neither the European Union nor the granting authority can be held responsible for them.
